# The Role of Adipose Tissue in Insulin Resistance in Women of African Ancestry

**DOI:** 10.1155/2013/952916

**Published:** 2013-01-14

**Authors:** Julia H. Goedecke, Naomi S. Levitt, Juliet Evans, Nicole Ellman, David John Hume, Liske Kotze, Mehreen Tootla, Hendriena Victor, Dheshnie Keswell

**Affiliations:** ^1^UCT/MRC Research Unit for Exercise Science and Sports Medicine, South African Medical Research Council, Parow, Cape Town 7505, South Africa; ^2^UCT/MRC Research Unit for Exercise Science and Sports Medicine, Department of Human Biology, Health Sciences Faculty, University of Cape Town, Cape Town 7725, South Africa; ^3^Endocrine Unit, Department of Medicine, University of Cape Town, Cape Town 8001, South Africa; ^4^Health Impact Assessment, Western Cape Department of Health, Cape Town 8001, South Africa

## Abstract

Women of African ancestry, particularly those living in industrialized countries, experience a disproportionately higher prevalence of type 2 diabetes (T2D) compared to their white counterparts. Similarly, obesity and insulin resistance, which are major risk factors for T2D, are greater in black compared to white women. The exact mechanisms underlying these phenomena are not known. This paper will focus on the role of adipose tissue biology. Firstly, the characteristic body fat distribution of women of African ancestry will be discussed, followed by the depot-specific associations with insulin resistance. Factors involved in adipose tissue biology and their relation to insulin sensitivity will then be explored, including the role of sex hormones, glucocorticoid metabolism, lipolysis and adipogenesis, and their consequent effects on adipose tissue hypoxia, oxidative stress, and inflammation. Finally the role of ectopic fat deposition will be discussed. The paper proposes directions for future research, in particular highlighting the need for longitudinal and/or intervention studies to better understand the mechanisms underlying the high prevalence of insulin resistance and T2D in women of African ancestry.

## 1. Introduction

The rising global burden of type 2 diabetes (T2D) is being borne primarily by low and middle income countries, including those in Africa, where there is recent evidence of a rising prevalence amongst urban black South Africans [1]. Further, people of African ancestry living in high income countries such as the United States of America (USA), United Kingdom, (UK) and Europe have a much higher prevalence of T2D than their respective countrymen and thereby also contribute to the increasing burden, albeit to a less extent [2]. The rising prevalence of T2D is being fuelled, in part, by the high rates of obesity in these communities. For example, 87% of all T2D in South Africa is attributable to elevated body mass index (BMI) [3] and obesity rates are higher in African diaspora communities compared to residents of the respective high income countries, particularly amongst women [2].

Insulin resistance, a common accompaniment of obesity, is a significant risk factor for T2D. However, there are also ethnic differences in the level of insulin resistance, with black South African women and African American women being more insulin resistant than their white counterparts, even when matched for age and BMI [4–6]. Insulin resistance in women of African ancestry is associated with an appropriately greater insulin response to maintain normoglycemia [4, 7]. The resultant hyperinsulinemia is due to an increase in insulin secretion as well as reduced hepatic clearance [4, 7]. The latter has been proposed as a potential mechanism to protect the beta cell from exhaustion [8], although it does not protect women of African ancestry from T2D. 

Although the exact mechanism is not known, there are numerous mechanisms that have been postulated to underlie the increased risk of T2D and insulin resistance in women of African ancestry. This paper will focus on the role of adipose tissue biology. The characteristic body fat distribution of women of African ancestry will be discussed first, followed by the depot-specific associations with insulin resistance, and then various biological factors within adipose tissue will be explored. As most studies have examined these topics by comparing black and white women, these comparative data are presented, and where available, the ethnic-specific associations with body fat distribution and insulin sensitivity are described. 

## 2. Body Fat Distribution

Traditionally one of the major determinants of insulin resistance is the centralization of body fat [9], more specifically increased visceral adipose tissue (VAT) [10], while peripheral (glutealfemoral) subcutaneous adipose tissue (SAT) deposition has been shown to be “protective” against insulin resistance (in predominately white populations) [11]. Yet, many studies in South Africa and the USA have shown that at the same level of BMI or waist circumference, black women are more insulin resistant than their white counterparts, despite having less VAT [4–6]. The major contributor to abdominal fat in African women is SAT, largely superficial rather than deep SAT [12, 13], and is accompanied by greater peripheral (gluteo-femoral) fat [14]. Thus, irrespective of the size of the depot, there is evidence of ethnic differences in the association between regional fat distribution and insulin sensitivity. In white women, VAT is the most significant determinant of insulin sensitivity [10], whereas in black women, insulin sensitivity is more closely associated with abdominal SAT than VAT [5, 12, 13]. In black South African women, this association is stronger for the deep SAT than the superficial SAT depot [12]. Further, we showed for the first time that gluteal SAT is negatively correlated with insulin sensitivity in black, but not white women [14].

Several mechanisms have been proposed for the depot-specific associations between adipose tissue accumulation and insulin sensitivity. Compared to SAT, VAT is more lipolytic [15], has greater 11**β**-hydroxysteroid dehydrogenase-1 (11HSD1) activity (which reactivates inactive cortisone to active cortisol) [16], and has reduced AMP-activated protein kinase (AMPK) levels [17] and a higher inflammatory profile [17, 18]. Notably, the products of VAT are released directly into the hepatic portal system. However, SAT, probably due to its sheer volume, accounts for ~80% of systemic circulating free fatty acids and produces large amounts of adipokines that have effects on the liver, skeletal muscle, and pancreas [19]. Abdominal deep SAT has been shown to be metabolically distinct to abdominal superficial SAT [20], displaying greater lipolytic activity [21], a lower intracellular pool of glucose transporter type 4 (GLUT4) [20], a higher inflammatory profile [22], and a more saturated fatty acid profile [23]. Gluteo-femoral SAT differs from abdominal SAT in that it has higher lipoprotein lipase activity [24], is less lipolytic [25], has larger adipocytes [26], and has consequently been proposed as a “metabolic sink,” trapping excess free fatty acids (FFAs) and protecting against ectopic fat deposition [27]. Pinnick et al. [28] recently demonstrated that gluteo-femoral SAT has other insulin-sensitizing effects, as it expresses higher levels of stearoyl-CoA desaturase (SCD1), the key enzyme involved in the desaturation of palmitic acid (16 : 0) to palmitoleic acid (16 : 1n-7).

Although many factors have been purported to influence body fat distribution and the association with insulin sensitivity, sex hormones are considered one of the most relevant.

## 3. Sex Hormones

The centralization of fat distribution, in particular increasing VAT, in estrogen deficient states such as menopause and postovariectomy, is well known [29, 30]. However, there is scanty evidence linking alterations in estrogen levels or exposure, to the characteristic fat distribution seen in women of African ancestry. For example, Casazza et al. [31] showed that African American girls had higher estradiol levels and reached menarche at a younger age than white American girls. Menarche was associated with accelerated weight gain in African American girls only, but the distribution of fat between the upper and lower body did not differ by ethnicity. Further, studies examining ethnic differences in hormone levels in pre-, peri- and postmenopausal women are conflicting, with studies showing higher, similar, or lower levels of estrogen in African American compared to white American women [32–34]. Differences in body fat and its distribution, as well as age, menopause type, and health behaviors, are important factors explaining the discrepancies in sex hormone levels between women of different ethnicities. It is, however, relevant to bear in mind that the effects of estrogen are mediated by binding to its receptors alpha (ER**α**) and beta (ER**β**), with their relative ratios regulating the biological response [35]. Murine adipose tissue ER**α** and ER**β** knockout models have highlighted the role of ERs in regulating adipose tissue deposition and insulin sensitivity [36, 37]. However, there is a limited research in humans and their contribution to the ethnic differences in body fat distribution and insulin sensitivity that remains to be explored. 

## 4. Adipose Glucocorticoid Metabolism

An additional factor that may underlie the ethnic-specific association between regional fat distribution and insulin resistance may be exposure to glucocorticoids. A case in point is the pathological condition of cortisol excess, Cushing's syndrome, which is characterized by central obesity, glucose intolerance, and insulin resistance [38]. Studies examining changes in the activity of the hypothalamic-pituitary-adrenal (HPA) axis with obesity have found that circulating levels of cortisol are normal, or even low [39]. However, glucocorticoid action on target tissues depends not only on the circulating concentrations, but also on the tissue sensitivity to glucocorticoids, which is dependent on 11HSD1, which converts inactive cortisone to active cortisol, and the glucocorticoid receptor-*α* (GR*α*). 11HSD1 is of potential pathogenic importance in body fat distribution and insulin resistance, since transgenic mice overexpressing 11HSD1 selectively in adipose tissue have increased VAT accumulation and insulin resistance [40]. In contrast, 11HSD1 knockout mice resist the development of insulin resistance, even on a high-fat diet [41]. In obese humans, 11HSD1 activity and mRNA levels are elevated in VAT [16] and abdominal SAT and associate with insulin resistance [16, 42]. 

In the absence of published studies that have compared glucocorticoid metabolism within adipose tissue depots in women of African ancestry, our preliminary data (Goedecke et al., unpublished) showed that abdominal and gluteal SAT 11HSD1 mRNA and activity increased with obesity, independent of ethnicity. In addition, in white but not black women, increased 11HSD1 activity in SAT was associated with increased VAT and reduced insulin sensitivity. On the other hand, abdominal and gluteal SAT GR*α* mRNA were down-regulated to a greater extent with obesity in black compared to white women. In black women only, reduced GR*α* mRNA in gluteal SAT correlated with reduced insulin sensitivity, possibly mediated via an associated increase in inflammatory gene expression and a decrease in peroxisomal proliferator-activated receptor-*γ* (PPAR-*γ*) and adiponectin expression. Intervention studies are required to gain a greater understanding of these findings.

## 5. Adipose Tissue Lipolysis

Many studies have examined ethnic differences in adipose tissue lipolysis in an attempt to explain the higher levels of insulin resistance in women of African ancestry compared to white women, matched for body fatness. The findings of these studies are, however, conflicting, showing lower, higher, and similar rates of lipolysis between obese black and white women, irrespective of whether the measurements were performed *in vivo* or *in vitro* (Table 1). Different techniques used to assess lipolysis, the age of the participants, the degree of glucose tolerance, and the ethnic differences in VAT and plasma insulin levels may explain these conflicting findings. Currently, however, there is no clear evidence to suggest that women of African ancestry have higher rates of lipolysis than their white counterparts, and that this might explain their disproportionately high prevalence of insulin resistance. Rather, differences in adipogenesis may be implicated.

## 6. Adipogenesis

Adipose tissue accumulation can occur either by hyperplasia (increased adipocyte number) or hypertrophy (increased adipocyte size), with the latter being more closely associated with insulin resistance [54]. Although the exact mechanism whereby hypertrophy exerts its effects on insulin signaling is not known, a number of mechanisms have been proposed, including, amongst others, reduced adipogenic potential, increased cellular hypoxia, and increased oxidative stress. 

We recently found an ethnic-specific association between obesity, adipogenesis, and insulin sensitivity [14]. Adipogenic and lipogenic genes were more highly expressed in the peripheral (gluteal) tissue of normal-weight black compared to normal-weight white women, but were downregulated to a greater extent in obese black versus obese white women, with the latter being associated with reduced insulin sensitivity in black, but not white women [14]. These findings have been confirmed in two separate studies. In a sample of normal-weight black and white South African women, adipogenesis was greater in preadipocytes isolated from the mammary gland tissue of black than white women [55]. In contrast, the expression of genes involved in adipogenesis including PPAR*γ*, SCD1, and lipin-1*β* was reduced in SAT of African American compared to white American women, who were on average obese [56]. Together, these results might suggest that reduced adipogenic potential may increase adipocyte hypertrophy leading to insulin resistance. Indeed, van Tienen et al. [57] showed the decreased expression of adipogenic genes in SAT preadipocytes isolated from T2D subjects compared to controls.

Earlier studies have shown larger adipocyte size in women of African ancestry compared to their white counterparts [58], and that gluteal (but not abdominal) adipocyte size correlated with insulin levels in postmenopausal African American women, but not white American women [26]. However, some researchers postulate that it is not the size of the adipocytes, but rather the distribution of cell size that may contribute to insulin resistance [59]. In preliminary results from our laboratory, black South African women had a larger median gluteal adipocyte size and a greater proportion of large gluteal adipocytes, while white women had a greater proportion of small cells (Keswell et al. unpublished). These data, together with our findings of negative correlation between gluteal fat mass and insulin sensitivity [14], suggest that central obesity may not be a good predictor of insulin resistance in women of African ancestry, but rather that gluteal adipocyte size may be a more sensitive indicator of insulin resistance. 

## 7. Hypoxia

Hypertrophy of adipocytes, particularly when the expanding cell mass exceeds the compensatory vascular supply, results in hypoxia in adipose tissue, with a concomitant reduction in insulin sensitivity [60, 61]. Cellular adaptation to hypoxia is accomplished through the activation of an array of oxygen sensing transcription factors, including hypoxia inducible factor-1 (HIF-1) [62], which induces the expression of pro-angiogenic proteins such as vascular endothelial growth factor (VEGF) and pyruvate dehydrogenase kinase-1 (PDK-1) [63]. However, in obese adipose tissue, even though HIF-1 protein levels increase, VEGF does not increase [64] and is accompanied by reduced capillary density [60, 64]. Furthermore, plasminogen activator inhibitor-1 (PAI-1), a stimulator of angiogenesis, is upregulated during obesity in VAT and SAT depots [65]. Notably, Festa et al. [66] showed that the expression of PAI-1 was lower in women of African ancestry compared to white and Hispanic women. We, therefore, speculate that higher levels of hypoxia may increase the expression of HIF-1 in adipose tissue and result in higher levels of inflammation and oxidative stress in African women. 

## 8. Oxidative Stress

Oxidative stress is the imbalance between the production of reactive oxygen species (ROS) and the cells antioxidant defense mechanisms that leads to the damage of proteins, lipids, and DNA. Although many studies have been performed in animal models to determine the association between oxidative stress and insulin resistance [67], human studies are limited. In healthy, nondiabetic women, increased protein carbonyls, a marker for oxidative stress, were associated with increased FFA and reduced insulin sensitivity in African Americans, but not European or Americans [68]. To our knowledge, no published studies have examined the association between oxidative stress within adipose tissue and insulin resistance, while a plethora of studies have examined the inflammatory profile of adipose tissue and its association with insulin resistance. 

## 9. Adipose Tissue Inflammation

Adipose tissue of obese individuals and patients with T2D is characterized by increased expression and/or secretion of several proinflammatory cytokines (e.g., tumor necrosis factor-*α* (TNF-*α*), interleukin (IL)-18, IL-6), chemokines (e.g., C-C motif ligand (CCL 2)), macrophage markers (e.g., CD68 and CD14), and adipokines (leptin), and decreased expression of the insulin-sensitizing adipokine, adiponectin [69]. Studies in humans have shown that individuals with inflamed abdominal SAT (characterized by increased gene expression of inflammatory proteins and presence of crown-like structures) are more hyperinsulinemic and insulin resistant compared to BMI-matched individuals without inflamed SAT [70]. 

A few studies have examined ethnic differences in the inflammatory profile of adipose tissue. In a sample of morbidly obese African American and white American women undergoing bariatric surgery, no ethnic differences in mRNA levels or *in vitro* release of IL-6, IL-8, and prostaglandin E_2_ from VAT were reported [71]. In contrast, our group found that black South African women had a higher abdominal and gluteal SAT inflammatory gene expression (characterized by increased CCL2 and its receptor CCR2, CD68, TNF-*α*, colony stimulating factor- (CSF-1) and macrophage inhibitory factor (MIF)) than white women, independent of age, total adiposity, and VAT [72]. Despite being more insulin resistant and having a higher inflammatory profile, SAT inflammatory gene expression only accounted for 20% of the variance in insulin sensitivity in black SA women, compared to 56% in white SA women [72]. In contrast to our findings, Smith et al. [56] found that the mRNA levels of abdominal SAT CD68, leptin, and retinol binding protein-4 were similar between African American and white American women, matched for BMI and insulin sensitivity. This variation in findings may be attributable to the fact that adipocytokines act in an endocrine, paracrine, and autocrine manner, making it difficult to capture their true effects using traditional molecular biology techniques. Furthermore, differences in age, body fatness, and level of insulin sensitivity may explain the disparate findings between the studies. 

With the exception of the adipocyte-derived proteins, adiponectin, and leptin, circulating levels of inflammatory proteins are largely derived from nonfat cells, including immune and endothelial cells within adipose tissue. As a result, ethnic differences in circulating levels of IL-6, IL-18, TNFR1, TNFR2, iCAM-1, VCAM-1, and adiponectin have largely been shown to be independent of the level of adiposity [73] (Table 2). However, in some instances, ethnic differences in circulating inflammatory proteins, including the acute phase protein, C-reactive protein (CRP), and the adipokine, leptin, have been attributed to differences in adiposity [49]. Whether or not other confounding variables may explain these differences remains to be explored.

Studies comparing the association between inflammatory markers and risk factors for T2D between ethnic groups have shown inconsistent results for some, but not all inflammatory proteins. For example, higher circulating levels of the insulin-sensitizing and anti-inflammatory hormone adiponectin were associated with a more favorable metabolic profile (lower BMI, insulin levels, and HOMA-IR) in white, but not black women [52, 53]. Similarly, circulating levels of the pro-inflammatory cytokine IL-6 have been shown to correlate with insulin sensitivity in overweight white American women and not overweight African American women [50]. The heterogeneity in the associations between ethnic groups highlights the need for prospective studies investigating the role of circulating inflammatory markers for risk prediction in different populations.

## 10. Ectopic Fat Deposition

A lower capacity to store fat in the periphery and higher rates of lipolysis is associated with a redirection of excess lipids to nonadipose depots, notably the liver, muscle, heart, and pancreas, where they accumulate as ectopic fat, which has been linked to insulin resistance [27, 74]. Ectopic fat deposition in the liver and skeletal muscle, measured using ^1^H magnetic resonance spectroscopy (MRS) and magnetic resonance imaging (MRI), was more tightly (negatively) correlated with systemic insulin sensitivity than was VAT volume [75]. However, this association does not hold true in all ethnic groups, as for the same level of body fat and/or insulin sensitivity, black women have less visceral and ectopic fat deposition than their white (or Hispanic) counterparts [5, 12, 76, 77] a phenomenon that is already present in black adolescent populations [78]. 

Hepatic steatosis (fatty liver) is associated with hepatic insulin resistance, characterized by decreased suppression of hepatic glucose production and lower insulin-stimulated liver glycogen synthesis. In a large multiethnic population-based study in the USA (*n* = 2, 287), Browning et al. [77], using MRS, showed that the prevalence of hepatic steatosis varied with ethnicity, with African Americans having a significantly lower prevalence than white and Hispanics (24% versus 33% and 45%, resp.), which could not be explained by ethnic differences in BMI, insulin sensitivity, or ethanol intake. Rather, that the ethnic differences in hepatic steatosis may be explained by the lower VAT in black compared to white women [76, 78]. This has also been found in a small study of obese premenopausal South Africa women matched for BMI and insulin sensitivity (Goedecke et al., unpublished). Thus, it appears that the relationship between hepatic steatosis and insulin sensitivity differs by ethnicity, with hepatic steatosis not being integral to the pathogenesis of insulin resistance in black women. 

On the other hand, intermuscular adipose tissue (IMAT) stores (measured using computerized tomography (CT) and MRI), a depot similar in size to VAT, were greater in African American compared to white individuals [79, 80] and were inversely associated with insulin sensitivity [79, 80]. However, when MRS has been used to quantify both intramyocellular (IMCL) and extramyocellular lipid (IMCL) content, only EMCL [81], and not IMCL [56, 81], was increased in African American and black South Africans (Goedecke et al., unpublished). Further, IMCL was associated with insulin sensitivity in white, but not black women and girls [81]. 

Increased skeletal muscle fat accumulation may be attributed to increased fatty acid availability and uptake and/or reduced fatty acid oxidation. Compared to their white counterparts, African American women have higher postabsorptive skeletal muscle lipoprotein lipase activity and are metabolically inflexible [82]. Further, *in vitro *studies have shown lower rates of skeletal muscle fatty acid oxidation [83], as well as reduced mitochondrial function in black compared to white premenopausal African American women [84]. Ethnic differences in the oxidative potential of the muscle may be related to muscle fiber composition, as African American women have been shown to have less type I oxidative fibers and more type IIb glycolytic fibers than their white counterparts [85]. The accumulation of by-products of fatty acid oxidation, such as diacylglycerol (DAG), ceramides, and long chain acyl-CoA, rather than IMAT alone, is associated with the development and progression of insulin resistance [86]. In addition, fat tissue releases many adipokines, including proinflammatory cytokines, as well as ROS that interfere with the insulin signaling pathway, thus impairing skeletal muscle glucose uptake. Further research is required to determine whether these by-products and adipokines explain the ethnic differences in insulin sensitivity.

## 11. Conclusion

This paper highlights the complexity of adipose tissue biology in the pathogenesis of insulin resistance and T2D in women. We have presented evidence that body fat distribution and adipose tissue biology and their association with insulin resistance differ by ethnicity. In summary, the research to date has shown that despite women of African ancestry being more insulin resistant than their white counterparts, they have less VAT and hepatic steatosis and more peripheral SAT. The larger SAT adipocyte size in women of African ancestry is associated with a reduced adipogenic capacity and a higher expression of inflammatory genes compared to their white counterparts. Given that most of the studies reviewed are cross-sectional in nature, causality cannot be inferred. Further, it is not known whether the ethnic differences in body composition can be explained by differences in SAT ER or GR expression, or whether adipose tissue hypertrophy in women of African ancestry is associated with increased hypoxia and/or oxidative stress in SAT and consequently insulin resistance. Longitudinal and interventional studies are critical for a better understanding of the mechanisms underlying the disproportionately high prevalence of insulin resistance and T2D in women of African ancestry.

## Figures and Tables

**Table 1:** Ethnic differences in adipose tissue lipolysis.

Participants	System	Ethnic differences in lipolysis	References
Nondiabetic, premenopausal, obese black and white US women	*In vivo *	Black < white for basal and insulin-suppressed systemic glycerol turnover, but not FFA turnover	Albu et al. [43]
Premenopausal, centrally obese black and white US women	*In vivo *	Black < white for basal and epinephrine-stimulated lipolysis	Racette et al. [44]
Morbidly obese black and white US women	*In vitro *	Black < white for basal lipolysis in VAT and SAT; black = white for isoproterenol-stimulated lipolysis, with and without insulin, in VAT and SAT	Barakat et al. [45]
Postmenopausal, overweight black and white US women	*In vitro *	Black > white for basal and insulin-inhibited lipolysis of abdominal SAT	Fried et al. [46]
Premenopausal, obese black and white South African (SA) women	*In vivo *	Black > white for basal and insulin-inhibited lipolysis of abdominal SAT	Van Der Merwe et al. [6]
Premenopausal, obese black and white SA women	*In vitro *	Black > white for insulin-inhibited lipolysis of abdominal SAT	Buthelezi et al. [47]
Premenopausal, normal-weight and obese black and white SA women	*In vivo *	Black = white for basal and insulin-suppressed lipolysis	Goedecke et al. [48]

**Table 2:** Ethnic differences in the relationship between circulating inflammatory markers and insulin resistance.

Inflammatory marker	Ethnic difference	Moderating factors	Association with risk	References
hsCRP	Black > white	BMI and SES attenuate the difference between ethnic groups	Significant association with IR in both black and white populations	Festa et al. [49]
IL-6	Black > white	Minor attenuation after adjusting for body fatness	Significant association with IR in white women only	Hyatt et al. [50]
IL-18	Black > white/Hispanic	Not defined	Significant associations with IR glucose in both black and white populations	Zirlik et al. [51]
Adiponectin	White > black	Still significant after adjustment for age, BMI, or WHR	Significant associations with IR in white women only	Ferris et al. [52]; Hulver et al. [53]

hsCRP: high-sensitivity C-reactive protein; BMI: body mass index; SES: socioeconomic status; IL: interleukin; IR: insulin resistance; WHR: waist-hip ratio.
